# Supplementary figures and images for: Vaginal microbiome-host interactions modeled in a human vagina-on-a-chip
Source: Microbiome. 2022 Nov 26;10:201. doi: 10.1186/s40168-022-01400-1 (PMC9701078; doi:10.1186/s40168-022-01400-1)

**
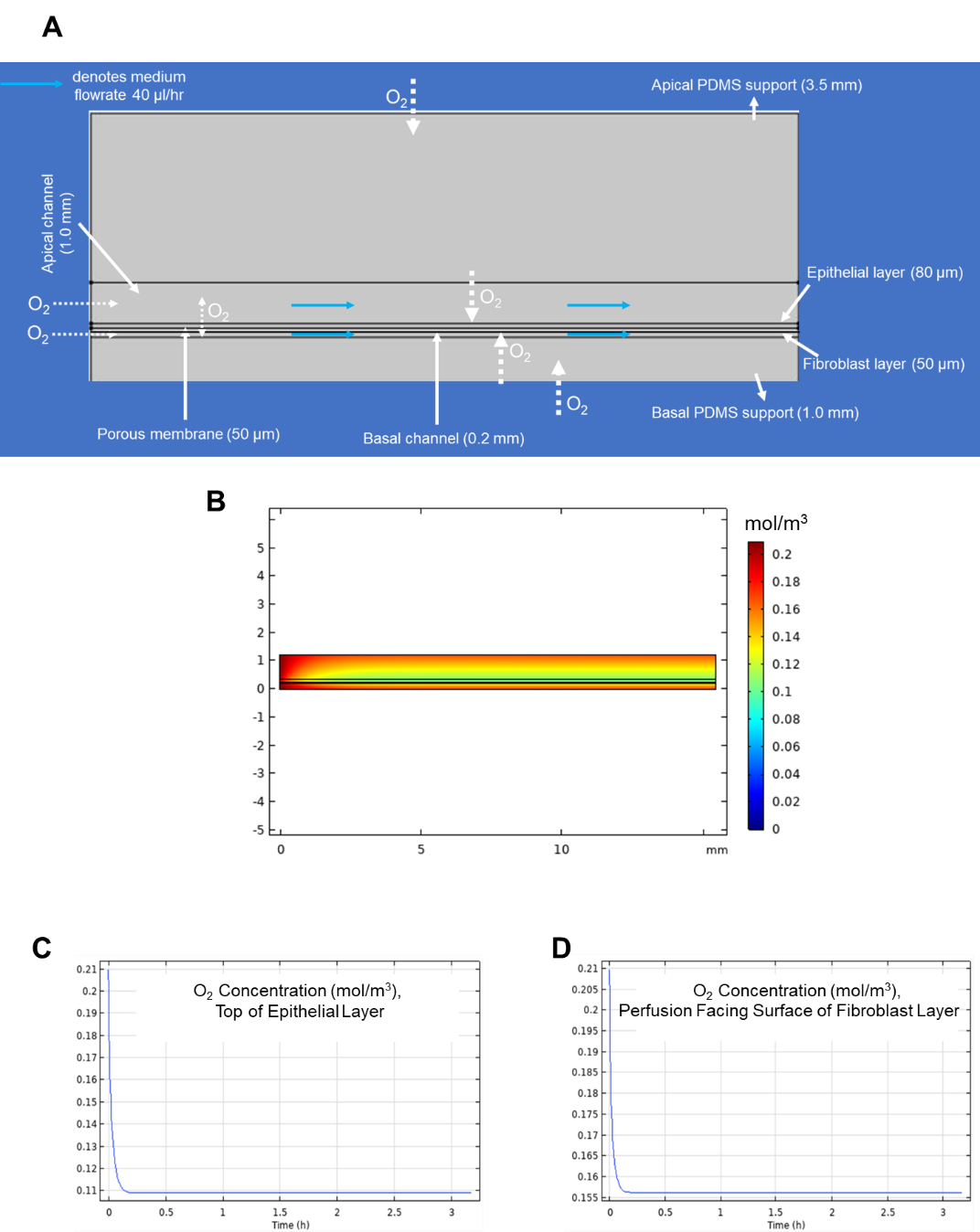
**

Supplement: Supplementary file 2 — Additional file 1: Supplementary Figure S1. Computational Model of Oxygen Gradient Generated by Vagina Chip. COMSOL 2D model with geometry adapted from the commercial Organ Chip (from Emulate Inc.) used in these studies. The chip contains two parallel channels under continuous flow with thick epithelial and fibroblast cell layers cultured respectively on the top and bottom of a 50 μm thick porous membrane that separates the two channels. Dotted arrows show sources of oxygen inflow and consumption in the chip. B) Surface plot demonstrating O2 distribution in the Vagina Chip. The lower graphs show results of O2 concentration simulations over time in apical epithelial channel (C) and basal fibroblast channel (D). [file 40168_2022_1400_MOESM1_ESM.docx]

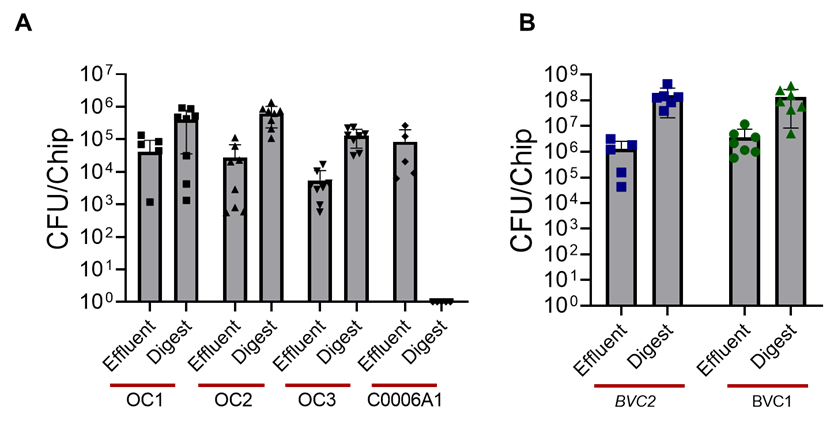


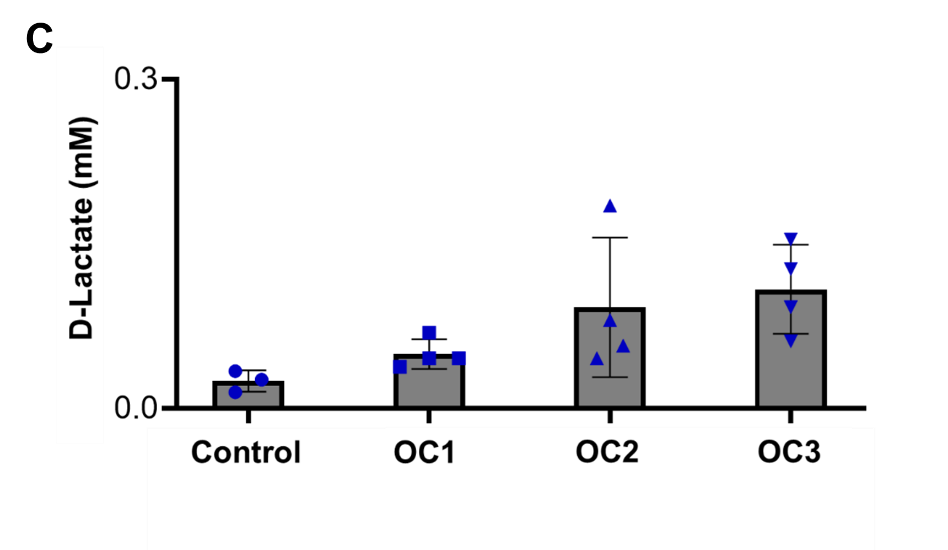

Supplement: Supplementary file 3 — Additional file 2: Supplementary Figure S2. Culture of L. crispatus and non-optimal G. vaginalis containing consortia in the Vagina Chip. A) CFU/Chip for L. crispatus is determined by quantifying non-adherent bacteria in effluents (total of 24, 48- and 72-hours) from the apical epithelial channel and viable bacteria adherent to the epithelium measured within tissue digests at 72-hours. B) CFU/Chip of BVC1 and BVC2 consortia bacteria measured in effluents (total of 24, 48- and 72-hours) from the vaginal epithelium-lined channel and epithelial tissue digest at 72-hours. C) D-lactate Production in Vagina Chips. D-lactate concentrations measured in effluents from the apical epithelial channel of chips cultured in the absence (Control) or presence of the OC1, OC2, or OC3 L. crispatus consortia collected at 24 hours post inoculation are shown; each data point indicates one chip. [file 40168_2022_1400_MOESM2_ESM.docx]
